# Discrete element method parameter calibration and validation of wheat based on the Tavares model

**DOI:** 10.1371/journal.pone.0346715

**Published:** 2026-04-13

**Authors:** Huapo Jia, Bo Feng, Wenbin Wu, Kexuan Yang

**Affiliations:** 1 School of Mechanical and Electrical Engineering, Henan University of Technology, Zhengzhou, Henan, China; 2 School of Mechanical Engineering, Zhengzhou University of Science and Technology, Zhengzhou, Henan, China; 3 COFCO Engineering (Henan) Engineering Equipment Co., Ltd., Kaifeng, Henan, China; Shandong University of Technology, CHINA

## Abstract

Existing discrete element studies on wheat predominantly utilize simplified models, which fail to accurately characterize differences in fracture behavior resulting from the anisotropic structure of wheat grains. Furthermore, there is a lack of systematic calibration for the core parameters of the crushing model employed in these studies. To address these issues, this study systematically calibrated and validated the contact parameters and crushing parameters of wheat grains using the Tavares crushing model. Employing a combined approach of physical experiments and simulation, contact parameters between wheat grains and the grinding roller surface material (white cast iron), as well as between wheat grains, were first determined. The accuracy of these parameters was validated via angle of repose tests. Subsequently, uniaxial compression tests and drop hammer impact tests were conducted to systematically calibrate the fracture energy distribution characteristics and cumulative damage coefficient for the Tavares model. Results indicate that: the elastic restitution coefficient, static friction coefficient, and rolling friction coefficient between wheat grains and white cast iron are 0.399, 0.426, and 0.179, respectively; the corresponding coefficients for wheat grain-wheat grain contact are 0.405, 0.322, and 0.039. The deviation between the simulated and experimental values of the angle of repose obtained using these parameters is only 1.05%. The fracture energy of wheat grains follows a log-normal distribution, with a median value of 2069.78 J/kg and a cumulative damage coefficient of 5. Substituting the calibrated parameters into the Tavares model enabled the establishment of a wheat compression-fracture simulation model that accounts for grain anisotropy. Quantitative comparison between the simulated and experimental compression force-displacement curves shows that the relative errors of the ensemble-averaged fracture force and fracture energy are 4.47% and 1.27%, respectively, providing objective quantitative validation for the calibrated model parameters. This study provides a theoretical foundation and practical tool for numerical simulation and process optimization in wheat roller milling, and offers significant reference value for improving wheat processing quality and efficiency.

## 1 Introduction

As a paramount staple food source in the dietary structure of the general population [1], wheat generates dozens of downstream products including wheat flour, bran, and cake flour, occupying a foundational role in the food processing industry chain [2]. In the conversion process from grain to end products, the crushing and grinding of wheat by roller mills is a critical step [3,4]. It directly determines flour yield, flour fineness, and nutrient retention. Excessive crushing can lead to bran particles becoming entrained in the flour, reducing its whiteness and quality and resulting in incomplete separation of the grains, reduced flour yield, and increased energy consumption in subsequent processing stages [5]. Therefore, precise analysis of the crushing patterns of wheat during roller milling is of significant practical importance for optimising processing techniques and improving product quality.

Conventional research methods face challenges in the real-time observation of microscopic mechanical behaviours during the crushing process, and the results have limited generalisability owing to variations in equipment parameters and material batches. The discrete element method (DEM), a powerful tool for particle dynamics analysis [6],can accurately simulate the particle crushing process under different material properties, loading conditions, and equipment structures [7].The essence of the DEM lies in treating the material not as a continuous medium, but rather in discretizing clusters of wheat grains into individual particles with distinct physical properties (mass, stiffness, frictional characteristics). By solving contact mechanics equations, this model simulates dynamic behaviors (collision, sliding, fragmentation).

Existing discrete element models for wheat research have inherent limitations, particularly in terms of adaptability to wheat-specific properties and model refinement. Wheat grains are inherently anisotropic materials: structurally consisting of endosperm, bran enclosed by multiple fibrous layers, and locally positioned embryos, exhibiting significant variations in density, elastic modulus, and other properties across these regions. The endosperm exhibits higher hardness than the bran. Mechanically, their compressive strength and fracture toughness vary along the long and short axes. Fracture along the short axis tends to occur within the endosperm, whereas tearing along the long axis is prone to affect the bran.

Existing mainstream discrete element model studies on wheat can be categorized into three types: (1) The Ab-T10 fracture model is only applicable to specific impact scenarios, fails to cope with the variable stress environment of roller milling, and overlooks the influence of anisotropy on fracture energy [8]; (2) Basic discrete element models focus on particle flow, simplify the crushing process, and fail to account for fatigue fracture following repeated loading of wheat grains [9–11]; (3) The Bonding model adopts fixed sub-particle generation rules, fails to replicate the non-uniform distribution of “large fragments (containing bran) and small fragments (endosperm),” and its parameters lack physical significance [12].

The Tavares fragmentation model, as a particle substitution model, has been extensively validated in the ore crushing field owing to its accurate characterization of the sub-particle substitution mechanism at the moment of particle fracture, fracture energy distribution characteristics, and damage accumulation effects [13,14]. In the agricultural sector, Li et al. [7] calibrated the Tavares model parameters specifically for cottonseed, whereas Shen et al. [15] calibrated those for rice grains. Owing to the ellipsoidal structure of wheat grains [16], their anisotropic mechanical properties [14,17,18], and complex internal endosperm-bran architecture [19], the application of the model in wheat fracture research remains limited. Systematic calibration of its key parameters to align with the material characteristics of wheat remains insufficient.

Building on the above, the overarching aim of this study is to develop and validate a Tavares-based compression fragmentation model for wheat that accounts for its anisotropic mechanical behaviour, thereby providing a numerical simulation tool for optimising wheat roller milling processes.

To achieve this aim, a multi-step parameter calibration approach was implemented. First, the contact parameters between wheat and the grinding roller material (white cast iron) were measured using custom-built mechatronic apparatus, while the inter-particle contact parameters were calibrated via angle of repose tests combined with Box-Behnken response surface methodology. Second, the core mechanical parameters of the Tavares model—including the median specific fracture energy (E₅₀), the standard deviation of fracture energy distribution (σ), the residual crushing energy (E∞), the characteristic microstructure size (d₀), and the damage accumulation coefficient (γ)—were systematically calibrated through uniaxial compression tests and drop-weight impact tests on wheat kernels of varying equivalent diameters. The fracture energy was characterised using a log-normal distribution to capture the inherent variability in wheat kernel strength, overcoming the limitations of the homogeneous material assumption inherent in previous models.

The reliability of the calibrated parameters was subsequently validated through multiple indicators: the angle of repose for inter-particle contact validation, and the compression force-displacement curves and fragment distribution patterns for Tavares model validation. By integrating these experimental and simulation approaches, this study elucidates the influence of wheat anisotropy on its fragmentation behaviour and establishes a foundation for numerical simulation and process optimisation in wheat roller milling.

## 2 Materials and methods

### 2.1 Tavares model

**Fig 1:**
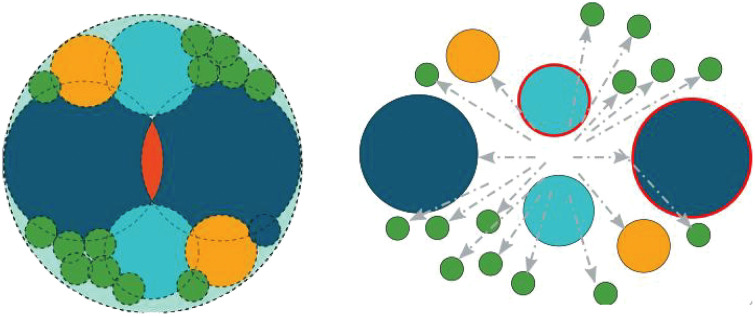
Schematic of spherical particle replacement.

The Tavares breakage model in Altair EDEM was employed to simulate the wheat roller crushing process. The Tavares breakage model is a particle-replacement model that can simulate the instantaneous fragmentation of particles under different stress intensities [14]. As shown in Fig 1, particle breakage is initiated when the impact energy (the ratio of the kinetic energy at impact to the particle mass) exceeds the fracture energy (the ratio of the energy absorbed during particle breakage to the particle mass). Once a particle fractured, it was immediately replaced by an array of smaller spheres.

In the Tavares fragmentation model, P(E), defined as the cumulative probability of wheat particle breakage at a given specific fracture energy E, follows a truncated log-normal distribution and is computed using Equations (1) and (2):


$$P(E)=\frac{\text{1}}{\text{2}}\left[\text{1}+\text{erf}\frac{\text{ln}\;E^{*}-\text{ln}\;E_{\text{50}}}{\sqrt{\text{2}}\sigma }\right]$$ (1)

$$E^{*}=\frac{E_{max}E}{E_{max}-E}$$ (2)

Where *P* (*E*) is the cumulative probability of wheat particle breakage at a given specific fracture energy; E^*^ is the relative specific fracture energy, J/kg; *E* is the specific fracture energy required for wheat particle breakage, J/kg; *E*_50_ is the median specific energy required for wheat particle breakage (the specific energy required for breakage with a 50% probability), J/kg; σ is the standard deviation of the log-normal distribution of the probability of wheat particle breakage; and *E*_max_ is the maximum specific fracture energy required for wheat particle breakage, J/kg.

The *E*_50_ for wheat particle breakage depends on the particle size. The relationship between *E*_50_ and the particle size is given by (3):


$$\begin{array}{c} E_{\text{50}}=\frac{E_{\infty }}{\text{1}+\frac{K_{\text{p}}}{K_{s}}}\left[\text{1}+{\left(\frac{d_{\text{0}}}{d_{\text{n}}}\right)}^{\varphi }\right] \end{array}$$ (3)

$$\begin{array}{c} K_{p}=\frac{Y_{p}}{\text{1}-v_{p}\text{2}} \end{array}$$ (4a)

$$\begin{array}{c} K_{s}=\frac{Y_{s}}{\text{1}-v_{s}\text{2}} \end{array}$$ (4b)

Eq (4a) is plane-strain modulus (stiffness) of wheat particles; Eq (4b) is plane-strain modulus (stiffness) of the grinding roller.

where *K*_p_ is the stiffness of wheat particles, N/m; *K*_s_ is the stiffness of the grinding roller, N/m; *E*_∞_ is the residual crushing energy of wheat particles, J/kg; *d*_0_ is the characteristic size of the microstructure of the material, mm; *d*_n_ is the particle size, mm; and φ is the power-law function of the *E*_50_ as a function of force;Yₚ and Yₛ are the Young's moduli of the wheat particle and roller material,Pa;νₚ and νₛ are their respective Poisson's ratios.

The *E*_∞_, *d*_0_, and φ values in Eq (3) can be fitted using experimental data. When the impact energy was less than the fracture energy of the particles, the particles sustained internal damage but remained unbroken. This damage is rooted in the continuous damage mechanics theory, as shown in Eq (5):


$$\begin{array}{c} \overset{\text{‘}}{\underset{f}{E}}=E_{f}\left(1-D\right) \end{array}$$ (5)

where $\overset{\text{‘}}{\underset{f}{E}}\;$ is the fracture energy after damage, J/kg; $E_{f\;}$ is the initial fracture energy, J/kg; and *D* is the degree of damage.

As the degree of damage increases, the fracture energy of the particles decreases. The relationship between the degree of damage D and the impact energy is governed by an implicit equation, given by Eq (6):


$$D={\left[\frac{2\gamma }{\left(2\gamma -\text{5}D+\text{5}\right)}\frac{eE_{\text{k}}}{E_{\text{f}}}\right]}^{\frac{2\gamma }{\text{5}}}$$ (6)

$$e=\frac{\text{1}}{\text{1}+\frac{K_{\text{p}}}{K_{\text{s}}}}$$ (7)

where γ is the cumulative damage coefficient; e is the collision energy ratio; and *E*_k_ is the impact energy, J/kg.

Note that *D* appears on both sides of Eq (6), making it an implicit equation that cannot be solved directly in closed form. Within the DEM framework, this equation is solved iteratively at each time step for particles subjected to sub-critical impacts. Starting from an initial assumption of *D* = 0, the damage value is updated progressively until convergence is achieved, following the procedure described in Tavares [14]. This iterative scheme captures the progressive weakening of particles under repeated loading below the fracture threshold.

When the impact energy of a particle substantially exceeded its fracture energy, it was replaced by progeny fragments. The degree of fragmentation can be expressed by t_10_, which represents the percentage of the total mass of sub-particles <1/10 of the original wheat particle mass relative to the total mass of the original wheat particle, and is calculated using Eqs (8) and (9):


$$t_{\text{10}}=\frac{m_{\text{0}}}{m_{\text{1}}}\times \text{100}\%$$ (8)

$$t_{\text{10}}=A\left[\text{1}-exp\left(-b\frac{eE_{k}}{E_{50}}\right)\right]$$ (9)

where $m_{\text{0}}$ is the mass of wheat after breakage, g; $m_{\text{1}}$ is the mass of wheat before breakage, g; and *A* and *b* are model parameters obtained by fitting the experimental data, where *A* is a general parameter [20].

### 2.2 Schematic of the experimental and simulation workflow

**Fig 2:**
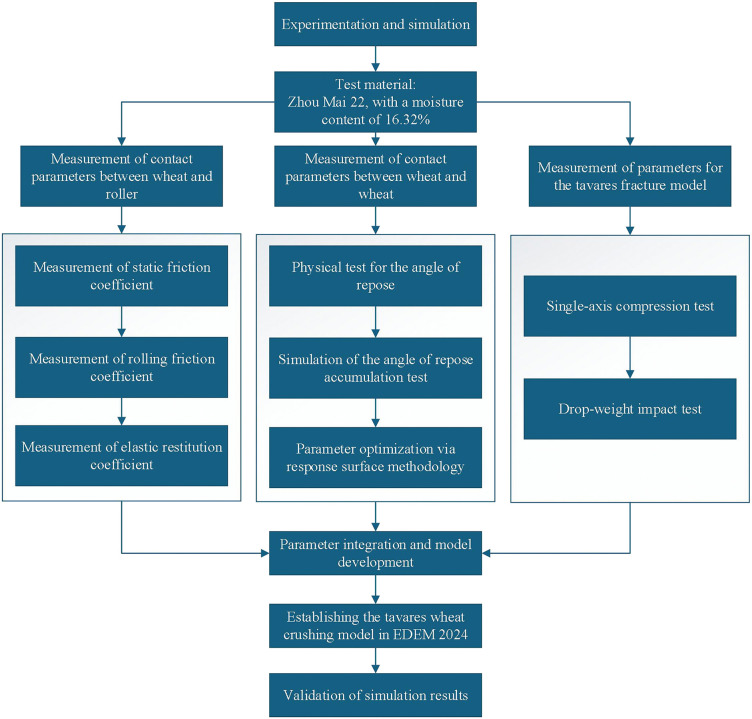
The schematic workflow of experimental and simulation steps.

Based on the experimental and simulation objectives of this study, common wheat varieties used in milling were selected to conduct measurement tests for contact parameters, including the static friction coefficient, rolling friction coefficient, and coefficient of restitution between wheat grains and white cast iron surfaces. The angle of repose test was employed to measure the contact parameters between wheat grains. Additionally, uniaxial compression tests and drop-weight impact tests were performed to determine the parameters for the Tavares breakage model. Using the obtained parameters, a Tavares breakage model for wheat was established in EDEM 2024. The simulation results were subsequently compared with actual wheat compression test results to verify the reliability of the model. An illustrative flowchart of the experimental, calibration, and validation processes is provided in Fig 2.

No specific permits were required for this study because the work involved discrete element method simulations and laboratory-based physical property measurements of commercially available wheat grains. It did not involve field research, endangered/protected species, or access to protected areas.

### 2.3 Experimental materials

The Zhoumai 22 wheat variety, produced in Henan Province, was selected as the study subject. In actual flour production, hard wheat must undergo moistening treatment before milling, with a target moisture content of 15.5% to 17.5%. After measuring the initial moisture content of wheat using an MB90 moisture meter, moistening was performed in accordance with the Agricultural Industry Standard of the People's Republic of China. After moistening, the moisture content of the wheat was found to be 16.32%.

### 2.4 Measurement experiment of contact parameters between wheat and white cast iron plate

#### 2.4.1 Measurement of static friction coefficient.

**Fig 3:**
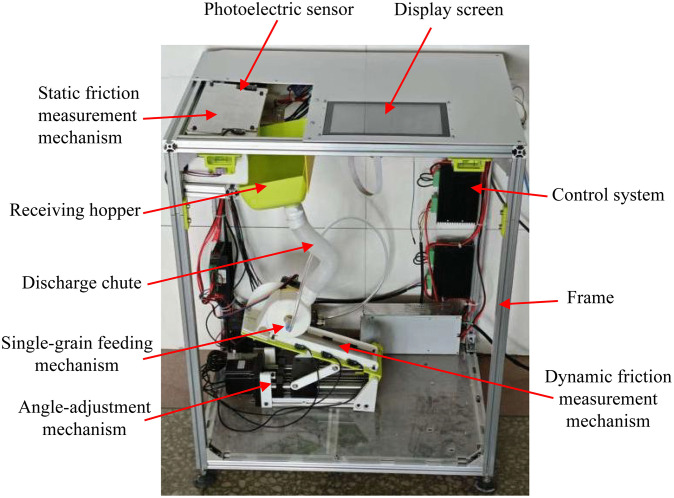
Grain friction coefficient measurement device.

The coefficient of friction is an important input parameter for simulating the wheat crushing process using the DEM [21]. Currently, several methods exist for measuring the coefficient of friction between grain particles and contact surfaces, including the inclined plane, tensile force, and rotating disc methods [22]. The inclined plane method features a simple measurement device and is easy to operate, making it one of the most commonly used methods for measuring the coefficient of friction. However, existing grain friction coefficient measurement methods based on the inclined plane method primarily rely on manual operation, requiring manual adjustment of the inclination angle until the particle begins to move, which is a cumbersome and time-consuming process. Additionally, the measurement process primarily relies on visual observation of particle sliding [23–25]. This renders large-scale particle measurement challenging (often requiring hundreds of particles), and only a small number of particle measurement results can be used for characterisation. However, plant particles exhibit significant individual differences, and a small sample size cannot reliably reflect the overall friction characteristics of the particles, leading to low accuracy of the measurement data [26]. To improve the measurement speed and accuracy of the static friction coefficient between wheat and white cast iron plates, a grain friction coefficient measurement device was developed (Fig 3). This device replaces traditional manual measurement methods that use a mechanical approach. Depending on the width of the static friction plate, multiple grains can be measured in a single test. During the measurement process, photoelectric sensors (Select the miniature infrared through-beam photoelectric sensor (EX-13EB) from Shenzhen Quansheng Sensing Technology Co., Ltd. Its minimum detectable particle size is 2 millimetres.) detected the movement of grains and transmitted signals to the control system of the device, which displayed the current tilt angle of the test plate and the static friction coefficient value in real time. Additionally, the control system of the device can count the number of particles falling at different angles during a single test and then calculate the weighted average of the measurement results, thereby improving the measurement speed and accuracy. Details regarding the measurement accuracy, repeatability and calibration of this apparatus are provided in reference [26].

**Fig 4:**
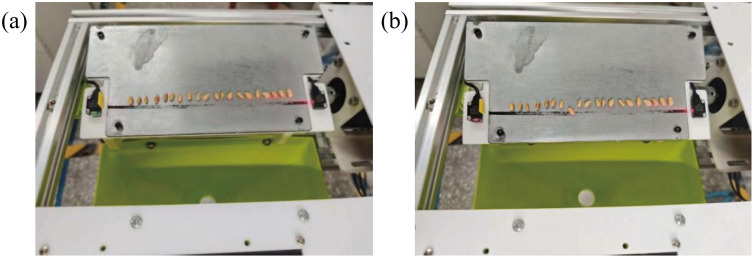
Static friction mechanism: (a) The arrangement of wheat grains at the start of the experiment; (b) The moment the wheat grains are captured by the photoelectric sensor.

A static friction coefficient measuring device was used to measure the static friction coefficient between the wheat and white cast iron plates. The measurement plate was then replaced with a white cast iron sample. After the measurement was initiated, the device was reset to a horizontal position. Subsequently, the wheat grains were placed on a white cast iron sample (above the sensor; Fig 4). At this point, the instrument is in a ready-to-test state, with the two photoelectric sensor indicator lights displaying red and green (Fig 4a). The motor drives the inclined plane to rotate clockwise, forming an increasing angle with the horizontal plane. When wheat grains begin to slide, the photoelectric sensors on both sides detect the sliding signal, and their indicator lights change from one red and one green to two red(Fig 4b). At this point, the system automatically records the inclination angle of the plane, static friction coefficient value, and number of falling wheat grains. Once all wheat grains have fallen off the measurement plate, the system sends a signal to reverse the rotation of the motor. The motor stopped rotating when the static friction measurement plane returned to the horizontal position. Because wheat grains are anisotropic, individual differences in shape and size can significantly affect test results. Therefore, to measure the static friction coefficient between wheat and white cast iron plates, this experiment was conducted in five groups, with 20 grains randomly selected per group from wheat with a moisture content of 16.32%, totaling 100 grains.

#### 2.4.2 Measurement of rolling friction coefficient.

**Fig 5:**
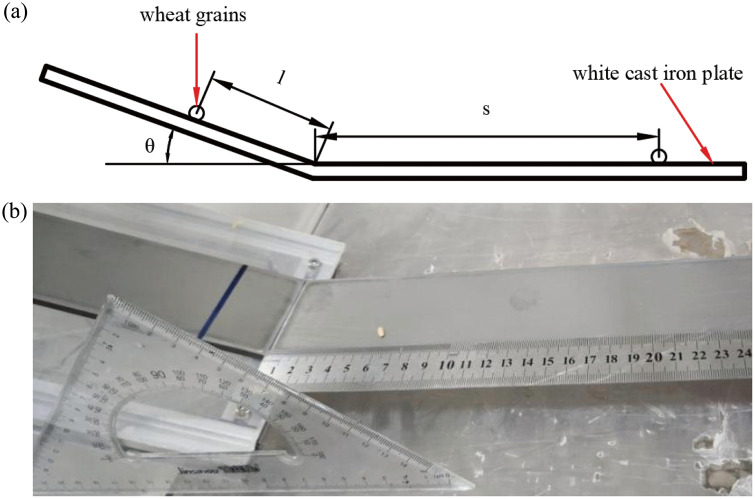
Rolling friction coefficient measurement test: (a) Measurement schematic diagram; (b) Actual measurement test.


Rolling friction refers to the rolling resistance generated by the deformation of the contact area when an object rolls or tends to roll on the surface of another object without sliding occurring during the process. Based on the static friction angle data between wheat and white cast iron plates and extensive preliminary experimental results [27], the inclination angle was set to 30°. At this angle, the wheat grains rolled on the inclined plane. The experimental setup for measuring the rolling friction coefficient is illustrated in Fig 5. Wheat was randomly divided into five groups with 20 grains per group. The wheat grains were placed at a starting position of *L* = 20 mm, and the maximum rolling distance *S* on the white cast iron plate was measured. The rolling friction coefficient was calculated using Eq (10):

$$\vartheta =\frac{l\;\text{sin}\;\theta }{l\;\text{cos }\theta +s}$$ (10)

where ϑis the rolling friction coefficient; θ is the slope angle, °; *l* is the slope length, mm; and *s* is the maximum ro*ll*ing distance of wheat particle, mm.

#### 2.4.3 Measurement of elastic restitution coefficient.

**Fig 6:**
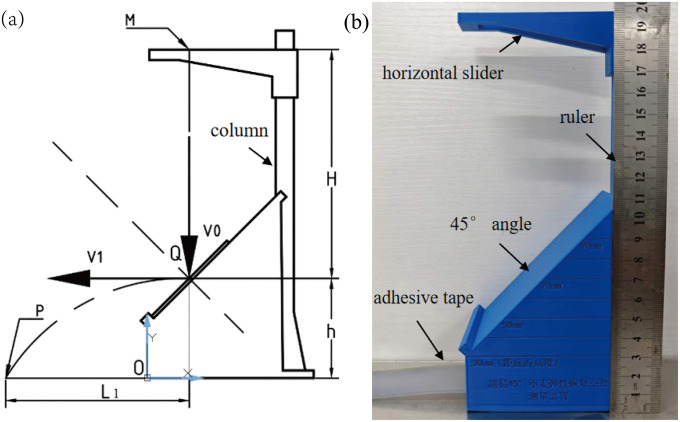
Device for measuring the coefficient of restitution: **(a)** Schematic diagram; **(b)** Physical setup.

The coefficient of elastic restitution is a key physical parameter used to measure the elastic deformation capacity of a material. Based on the principle of kinematic equations, this study developed a simple elastic restitution measuring device to measure the restitution coefficient between wheat and white cast iron plates (Fig 6). This apparatus is an improved version based on the wheat coefficient of restitution testing device proposed in Literature [28]. Its core principle and the computational logic of the kinematic equations remain unchanged. The original device's methodological validity has been verified through multiple sets of experiments. In this study, the drop-point positioning structure was optimised from the original design. Specifically, adhesive tape was used instead of the grease employed in Literature [28] to reduce lateral deviation of the wheat kernels during descent, thereby further ensuring consistency in test conditions. The repeatability and reliability of the apparatus inherit the well-established characteristics of the original method.

The inclined plane of the device had an angle of 45° and was designed to place the white cast iron sample. The upper part of the vertical column features grooves that align with the horizontal slider and is equipped with horizontal and vertical scales to facilitate measurements. During the experiment, the horizontal slider is adjusted to the set height, allowing the wheat grains to freely fall from point M. Under ideal conditions, a wheat grain, with an initial velocity V₀, collides with the sample at point Q and then rebounds horizontally at velocity *V*₁, ultimately landing at point P. According to the definition, the coefficient of elastic restitution is the ratio of the velocity at the end of the collision to the velocity at the beginning of the collision (i.e., *e*_1_ = *V*₁/*V*₀).

Owing to the irregular shape of the wheat grains, collisions may result in velocity components perpendicular to the paper surface (inward or outwards). To ensure data validity, the device was fitted with adhesive tape strips along the centreline of the landing plane. Grains landing on the tape had negligible out-of-plane velocity components, and only the effective collision data from grains landing on the tape were collected. The device was used to conduct three sets of tests at various drop heights. For each height, testing proceeded until 50 valid data points were obtained and the average was calculated.The coordinates of point M for the three test groups are (40, 200), (30, 200), and (20, 200).

By measuring the drop height and horizontal rebound displacement of wheat, combined with Eq (11), the coefficient of elastic restitution between the wheat and tooth roller surface material can be calculated [28]:


$$\begin{array}{c} e_{\text{1}}=\frac{V_{\text{1}}}{V_{\text{0}}}=\frac{L_{\text{1}}\text{cos}\;\text{45}\circ \sqrt{\text{g}/\text{2h}}}{\text{cos}\;\text{45}\circ \sqrt{\text{2g}H}}=\frac{L_{\text{1}}}{\text{2}\sqrt{H\text{h}}} \end{array}$$ (11)

where *e*_1_ is the coefficient of elastic restitution of wheat; *V*_0_ is the velocity of the wheat grains before colliding with the material, m/s; *V*_1_ is the velocity of the wheat grains after colliding with the material, m/s; *L*_1_ is the horizontal distance between the wheat landing point and collision point, m; *H* is the vertical distance between the landing point and collision point, m; and *h* is the vertical distance between the collision point and wheat landing point, m.

### 2.5 Calibration of contact parameters between wheat particles

#### 2.5.1 Angle of repose experiment.

The angle of repose characterises the angle between the free surface of the bulk material in the gravitational field and the horizontal plane when it reaches a state of equilibrium. The larger the angle, the greater the internal friction of the material and the poorer its dispersibility. When the wheat is stable, the component of gravity along the slope is less than or equal to the internal frictional force of the material. The contact parameters between the wheat particles can be determined by measuring the angle of repose.

**Fig 7:**
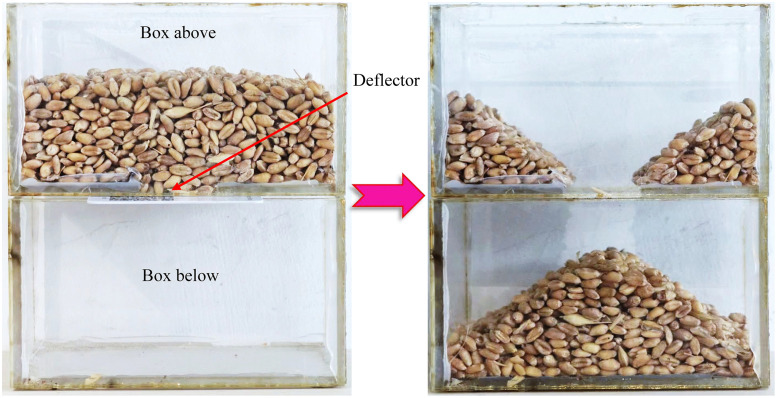
Angle of repose measurement apparatus and experiment.

A measuring device was fabricated to measure the angle of repose of bulk wheat (Fig 7). The device comprises two identical open rectangular containers. The bottom of the upper container had a rectangular discharge opening with a removable baffle inserted. During the experiment, wheat grains were added to the upper container at a predetermined height, and the container was gently shaken to ensure that the upper surface was level. The baffle was quickly removed to allow the wheat grains to flow freely into the lower container. Once the flow stopped, stable piles were formed in both containers and the angle of repose (between the piles and horizontal plane) was measured. The experiment was repeated 10 times.

#### 2.5.2 Simulation testing.

**Fig 8:**
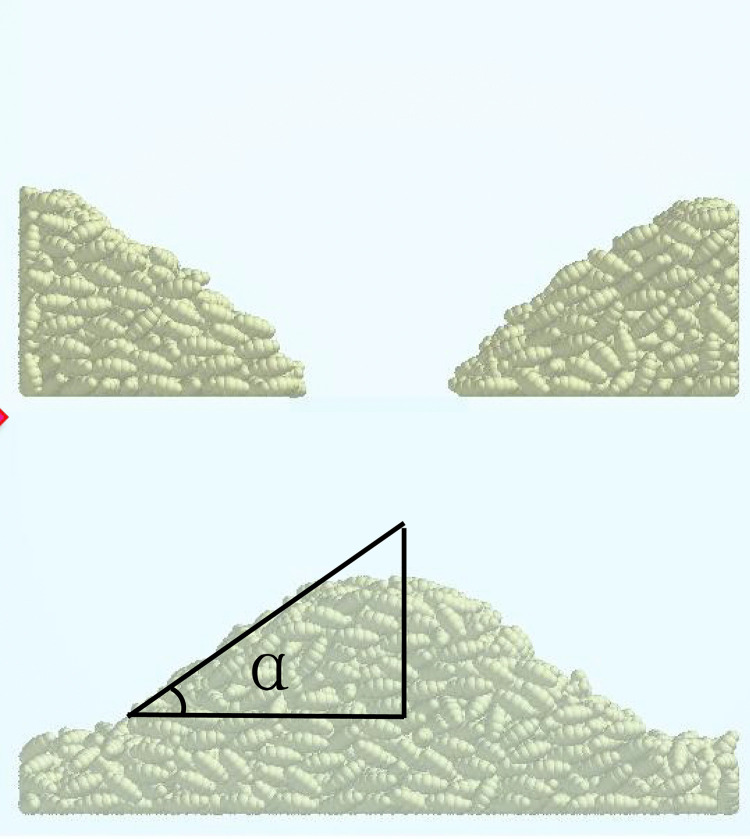
Simulation test of wheat angle of repose.

A simulation was conducted to validate obtained from the actual tests. Using a vernier calliper, the length (*L*), width (*W*), and thickness (*T*) of 60 wheat grains were randomly measured, resulting in an average length, width, and thickness of 6.35 mm, 3.50 mm, and 2.83 mm, respectively, for this batch of wheat. A wheat discrete element model was established based on three-dimensional measurements and the shape characteristics of wheat grains. An angle of repose measurement device was set up in the Altair EDEM software, with a particle factory installed at the top of the upper box. The particle factory generated 50 g of wheat particles to fill the upper box. Once the wheat particles had stabilised, a plate was withdrawn at a speed of 1 m/s until the wheat grains fell and stabilised, at which point the angle of repose was measured [29]. The simulation results for the angle of repose are shown in Fig 8. After the angle of repose experiment, the experimental images were processed and the angle of repose was measured.

### 2.6 Calibration test of wheat Tavares crushing model parameters

#### 2.6.1 Single-axis compression test of wheat particles.

Uniaxial compression tests were conducted on wheat with a moisture content of 16.32%. Prior to the compression tests, the three-axis dimensions and mass of wheat were measured using a vernier calliper and an Hochoice Model HC3204 electronic analytical balance, respectively. The wheat had an elliptical shape. During statistical analysis, the equivalent diameter of wheat was calculated using Eq (12) based on the isovolumetric method [30]:


$$D_{e}={\left(\frac{L\times {\left(W+T\right)}^{2}}{4}\right)}^{\frac{1}{3}}$$ (12)

where, *D*_e_ is the equivalent diameter of the wheat, mm; *L* is the length of wheat, mm; *W* is the width of wheat, mm; *T* is the thickness of wheat, mm.

**Fig 9:**
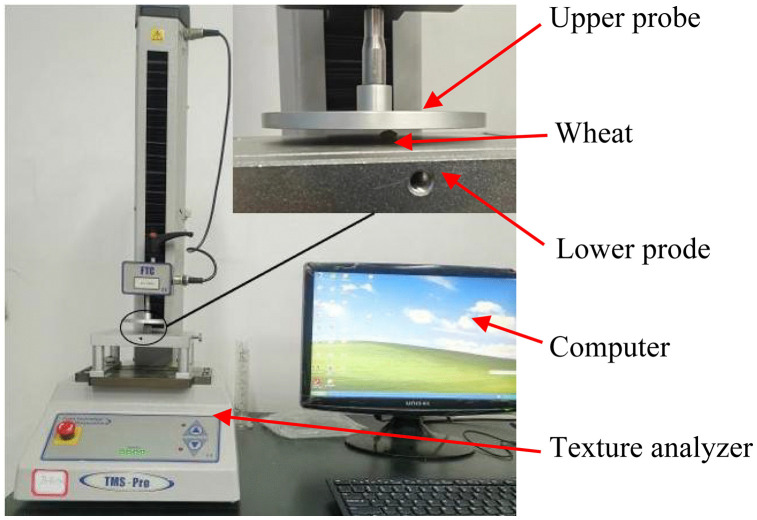
Wheat particle compression test.

Four equivalent diameter grades (3.68 mm, 4.01 mm, 4.28 mm, and 4.44 mm) were classified based on the isovolumetric equivalent diameter of wheat kernels. A total of 80 wheat grains with intact surfaces were selected for uniaxial compression tests, with the number of samples for each diameter grade determined according to the natural size distribution of the tested wheat batch (12, 22, 26, and 20 grains for the 3.68 mm, 4.01 mm, 4.28 mm, and 4.44 mm grades, respectively). Tests were performed using a TMS-PRO texture analyser to obtain the compression force-displacement curve of wheat grains during loading (Fig 9). The triaxial dimensions, mass, and compression force-displacement curve of each wheat grain were recorded in association with each other. During the test, the probe of the analyser was set to apply force at a speed of 15 mm/min.

#### 2.6.2 Wheat particle drop-weight impact test.

**Fig 10:**
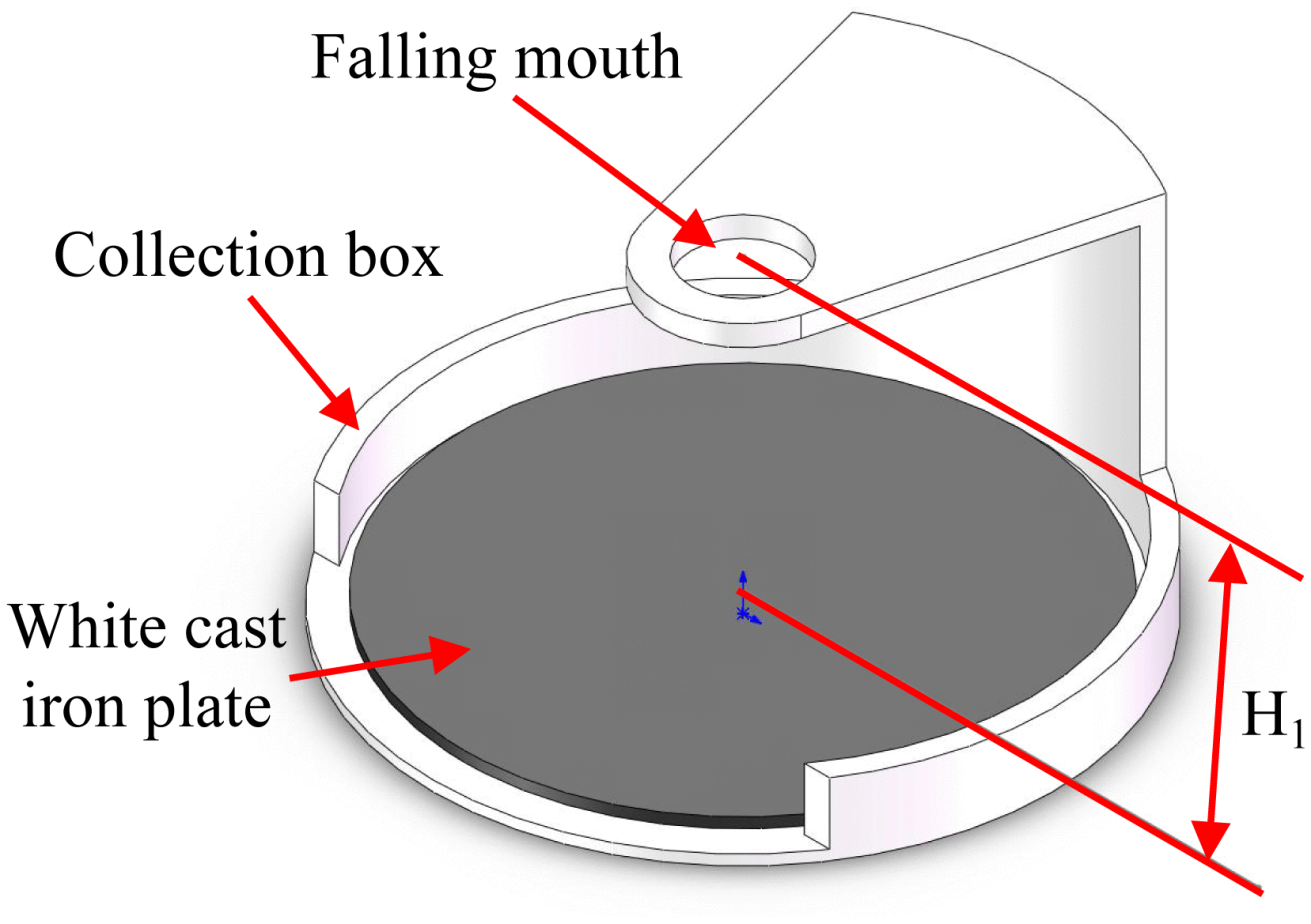
Drop-weight test apparatus.

To conduct the wheat particle drop-weight test, we developed a set of experimental equipment, the core components of which included steel balls, drop ports, collection boxes, and white cast iron plates (Fig 10). This apparatus was modified with reference to the test equipment for paddy drop-weight testing constructed by Shen et al. [15], with the core experimental principle (free-fall impact of a steel ball on the grain) remaining unchanged. By ensuring the coaxial alignment of the fixed drop port and the collection box, consistency in the steel ball’s impact point is maintained. Wheat kernels are placed at the centre of the collection box, a steel ball of specific mass is selected and the drop height is set. The ball is released for free-fall impact, and video recording is used to confirm the validity of the impact. The impact operation is repeated until the wheat kernel fractures, recording the number of drop-weight impacts at fracture and the corresponding impact energy. To enhance test stability, the fixing structure of the apparatus was optimised in this study to reduce equipment vibration during testing, ensuring uniform impact conditions for each trial and thereby guaranteeing the repeatability of the experimental results.

The drop port was fixed directly above the collection box to ensure that the steel balls landed at the same position. A mobile phone video-recording function was used to ensure the validity of the data. After each drop of the steel ball, the video was reviewed, and when the steel ball struck the wheat grain, it was recorded as a valid count.

During the test, the impact energy was set below the median energy value of E_50_. For each test, the wheat grain was repositioned at the centre of the collection box, and multiple drops were repeated until the wheat grain broke (air resistance was ignored in the impact test).

After completing multiple sets of repeated experiments, the number of steel ball drops causing wheat grain breakage was compiled, and the data were processed and analysed to determine the probability of wheat grain breakage under different numbers of drops.

When wheat particles were struck by steel balls, the impact energy was calculated using Eq (13):


$$E_{k}=\frac{m_{b}\overset{2}{\underset{0}{v}}e}{2m_{0}}$$ (13)

$$v_{\text{0}}=\sqrt{\text{2}gH_{\text{1}}}$$ (14)

where *m*_b_ is the steel ball mass, g; *V*_0_ is the steel ball impact velocity, m/s; and *H*_1_ is the height of the steel ball drop height, m.

To measure the cumulative damage coefficient, this study repeatedly impacted wheat particles using an impact energy of 268.8 J/kg (*m*_b_ = 32 g, *H*_1_ = 0.03 m, *m*_0_ = 0.035 g, g=9.8 m/s²).

## 3 Results

### 3.1 Calibration of wheat–white-cast-iron and wheat–wheat contact parameters

#### 3.1.1 Contact parameters between wheat and white cast iron plates.

The measured values of the static friction coefficient, rolling friction coefficient, and elastic restitution coefficient between wheat grains and white cast iron plates are listed in Table 1. The mean value of static friction coefficients of each group ranges from 0.405 ± 0.082 to 0.442 ± 0.097. The overall mean across all five groups was 0.426. The measured rolling friction coefficients yielded mean values ranges from 0.177 ± 0.045 to 0.181 ± 0.038. The overall mean across all five groups was 0.179. The mean elastic restitution coefficients at three different drop heights were 0.404 ± 0.017, 0.398 ± 0.016, and 0.395 ± 0.016, respectively. The overall mean for the three groups was 0.399.

**Table 1:** Test results of contact parameters between of wheat and white cast iron plate.

Contact parameters	Project	Group						
		1	2		3	4		5
Static friction coefficient	Mean value of each group	0.425	0.419		0.438	0.405		0.442
	Standard deviation (±)	0.082	0.077		0.089	0.082		0.097
	The mean values of 5 groups	0.426						
Rolling friction coefficient	Mean value of each group	0.177	0.179		0.180	0.179		0.181
	Standard deviation (±)	0.045	0.036		0.032	0.037		0.038
	The mean values of 5 groups	0.179						
Coefficients of elastic restitution	Mean value of each group	0.404		0.398			0.395	
	Standard deviation (±)	0.017		0.016			0.016	
	The mean values of 3 groups	0.399						

#### 3.1.2 Contact parameters between wheat particles.

**Fig 11:**
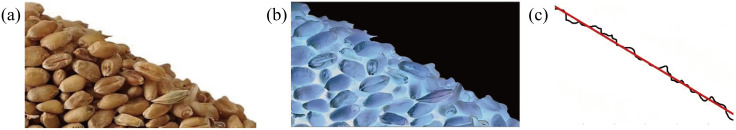
Image processing results for wheat angle of repose: (a) Original image of wheat grain piling;(b) Binarized image; (c) Angle of repose fitting curve.

The image processing results after the angle of repose test was completed are shown in Fig 11. Fig 11a shows the original image, and Fig 11b shows the image obtained after binarisation. The angle between the fitting curve in Fig 11c and the horizontal direction was the measured angle of repose. The average of the experimental data for the angle of repose test (Table 2) was calculated to determine an angle of repose of 29.765°.

**Table 2:** Test results of angle of repose.

Project	Group									
	1	2	3	4	5	6	7	8	9	10
Angle of repose (°)	28.156	29.705	28.937	29.314	28.563	31.892	30.641	31.208	30.982	30.401
Mean values (°)	29.765									

According to [31], the parameter ranges for the wheat-wheat coefficient of elastic restitution c_1_, the wheat-wheat static friction coefficient c_2_, and the wheat-wheat rolling friction coefficient c_3_ are 0.2 to 0.6, 0.25 to 0.45, and 0.02 to 0.06, respectively. The factors and levels of the simulation tests for the angle of repose of the accumulation in this study are listed in Table 3. Using Design-Expert 13.0 software, a three-factor, three-level response surface experiment based on the Box-Behnken design was conducted, with the simulated angle of repose as the response variable. Five central points were included, resulting in a total of 17 simulated trials. The experimental design and results are summarized in Table 4.

**Table 3:** Calibration test factors for wheat inter-particle parameters.

Factor	Level	
	Low-level (−1)	High-level (1)
Coefficient of elastic restitution (c_1_)	0.2	0.6
Coefficient of static friction (c_2_)	0.25	0.45
Coefficient of rolling friction (c_3_)	0.02	0.06

**Table 4:** Box–Behnken experimental design table and results for wheat angle of repose α.

Group	c_1_	c_2_	c_3_	α (°)	Group	c_1_	c_2_	c_3_	α (°)
1	0.2	0.25	0.04	29.780	10	0.4	0.45	0.02	28.920
2	0.6	0.25	0.04	30.104	11	0.4	0.25	0.06	31.801
3	0.2	0.45	0.04	32.936	12	0.4	0.45	0.06	34.353
4	0.6	0.45	0.04	31.549	13	0.4	0.35	0.04	31.697
5	0.2	0.35	0.02	28.645	14	0.4	0.35	0.04	31.740
6	0.6	0.35	0.02	28.403	15	0.4	0.35	0.04	32.059
7	0.2	0.35	0.06	34.803	16	0.4	0.35	0.04	31.844
8	0.6	0.35	0.06	32.705	17	0.4	0.35	0.04	31.779
9	0.4	0.25	0.02	26.752	\	\	\	\	\

To evaluate the stability and repeatability of the simulation tests, five replicate simulations were conducted at the central points of the response surface design (c₁ = 0.4, c₂ = 0.35, c₃ = 0.4) (Groups 13–17 in Table 4). The simulated angle of repose values for these five replicates were 31.697°, 31.740°, 32.059°, 31.844°, and 31.779°, respectively, with a mean of 31.82° and a standard deviation of 0.14°. The small standard deviation (0.14°) indicates that the reliability of the simulation model is relatively good. The variability from the central-point tests can be considered negligible in its influence on the response surface analysis results.

A second-order regression equation was obtained by analysing the test results to describe the influence of the coefficient of elastic restitution, static friction coefficient, and rolling friction coefficient between the wheat grains on the angle of repose of the wheat piles. The regression model is given by Eq (15). The ANOVA results for the regression equations are listed in Table 5.

**Table 5:** Analysis of variance for the angle of repose (response variable:α, unit: °) regression model.

Source	DOF	Quadratic sum	Mean square	F value	P value
Model	9	72.98	8.11	199.01	<0.0001
c_1_	1	1.45	1.45	35.56	0.0006
c_2_	1	10.86	10.86	266.61	<0.0001
c_3_	1	54.83	54.83	1345.62	<0.0001
c_1_ c_2_	1	0.7307	0.7307	17.93	0.0039
c_1_ c_3_	1	0.8613	0.8613	21.14	0.0025
c_2_ c_3_	1	0.0369	0.0369	0.9050	0.3731
c_1_^2^	1	0.0026	0.0026	0.0637	0.8080
c_2_^2^	1	2.11	2.11	51.71	0.0002
c_3_^2^	1	1.84	1.84	45.15	0.0003
Residual	7	0.2852	0.0407	/	/
Lack of fit	3	0.2054	0.0685	3.43	0.1323
Pure error	4	0.0798	0.0200	/	/
Total	16	73.26	/	/	/

Note: The regression model is defined in Eq (15). c₁, c₂, and c₃ represent the wheat-wheat coefficient of elastic restitution, static friction coefficient, and rolling friction coefficient, respectively. DOF: degrees of freedom. P < 0.01 indicates extremely significant; 0.01 ≤ P < 0.05 indicates significant.


$$\begin{array}{cc} \alpha =\; & 7.7673+10.48874{\text{c}}_{1}+67.79909{\text{c}}_{2}+29.268994{\text{c}}_{3}-21.37{\text{c}}_{1}{\text{c}}_{2}-116.00398{\text{c}}_{1}{\text{c}}_{3}+48.00625{\text{c}}_{2}{\text{c}}_{3}\\ & -0.620813{\text{c}}_{1}2-70.74075{\text{c}}_{2}2-165.2425{\text{c}}_{3}2 \end{array}$$ (15)

The regression model for the angle of repose was highly significant, with an F-value of 199.01 and P < 0.0001; the lack-of-fit term was non-significant (P = 0.1323), indicating a good fit of the model to the experimental data. The statistical results for individual factors and interaction terms were as follows: the coefficient of elastic restitution (c₁, F = 35.56, P = 0.0006), static friction coefficient (c₂, F = 266.61, P < 0.0001) and rolling friction coefficient (c₃, F = 1345.62, P < 0.0001) all exhibited significant effects on the angle of repose. For the interaction terms, c₁ × c₂ (F = 17.93, P = 0.0039) and c₁ × c₃ (F = 21.14, P = 0.0025) were significant, while c₂ × c₃ (F = 0.9050, P = 0.3731) was non-significant. Among the quadratic terms, c₂² (F = 51.71, P = 0.0002) and c₃² (F = 45.15, P = 0.0003) were highly significant, whereas c₁² (F = 0.0637, P = 0.8080) showed no significant effect.

**Fig 12:**
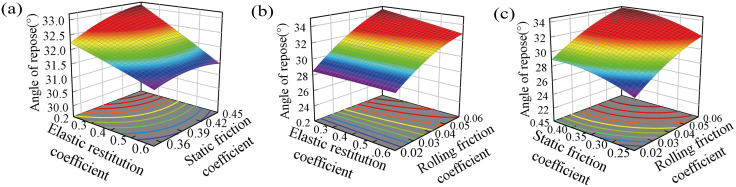
The interaction response surface of the influence of wheat-wheat contact parameters on the repose angle: (a) Elastic restitution coefficient and static friction coefficient; (b) Elastic restitution coefficient and rolling friction coefficient; (c) Static friction coefficient and rolling friction coefficient.

Fig 12 shows the interactive response surface diagram of the repose angle. Using the regression equation (Eq 15) and the response surface optimization module, the optimal wheat-wheat contact parameters were identified as: coefficient of elastic restitution = 0.405, static friction coefficient = 0.322, and rolling friction coefficient = 0.039. Five replicate angle of repose simulations were performed with the above parameters, resulting in a mean simulated angle of repose of 30.078° (standard deviation = 0.32°). The experimental mean angle of repose was 29.765° (standard deviation = 1.12°, n = 10 replicates), with a relative error of 1.05% between simulated and experimental values.

### 3.2 Tavares crushing model for wheat

#### 3.2.1 Fracture energy of different-sized wheat grains.

**Fig 13:**
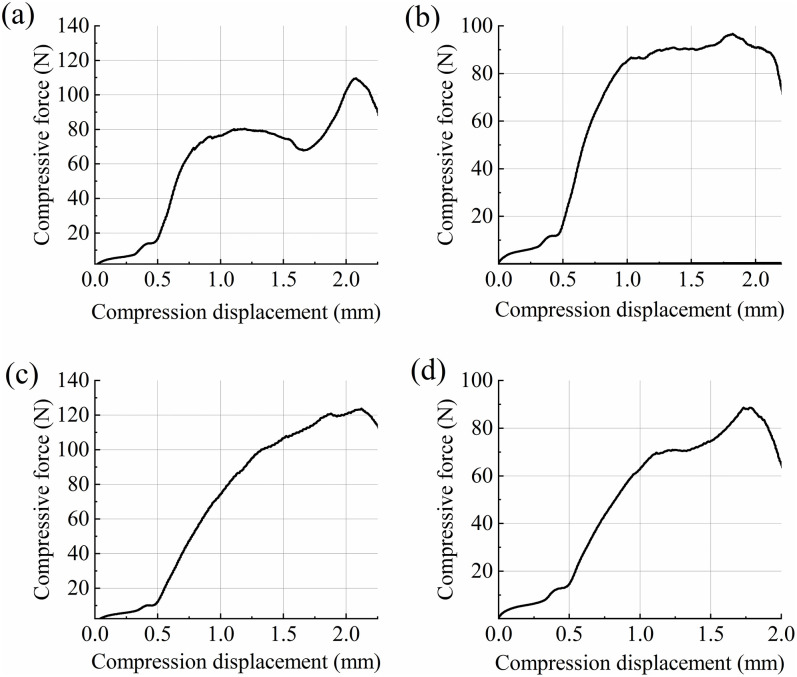
The average force-displacement curves of the compression processes of different equivalent diameter wheat grains: (a) 3.68 mm; (b) 4.01 mm; (c) 4.28 mm; (d) 4.44 mm.

**Fig 14:**
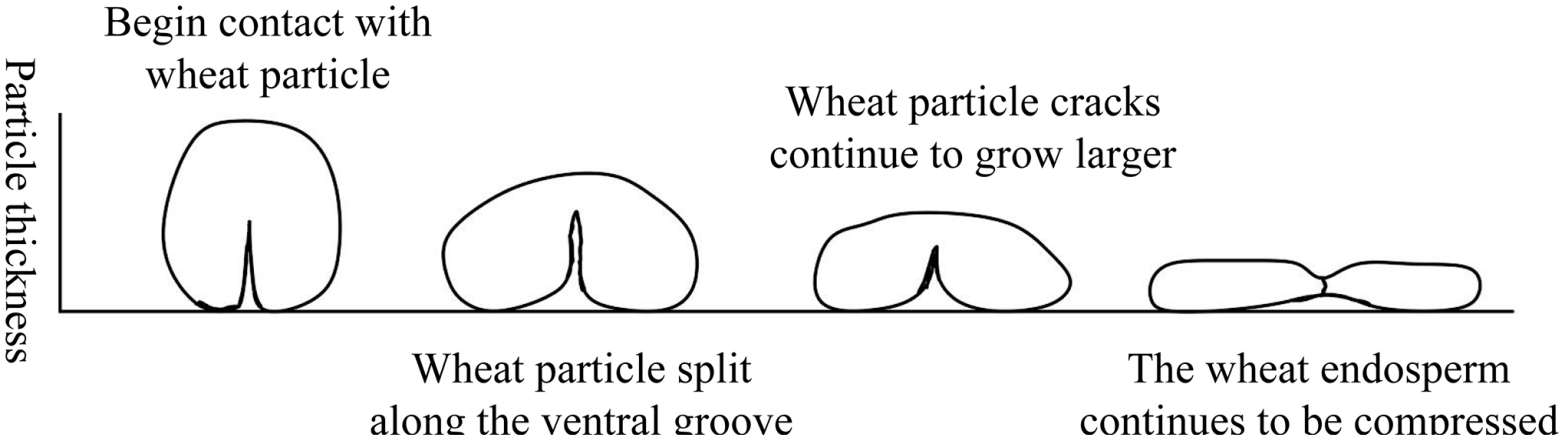
Wheat compression characteristics.

Four equivalent diameter grades were selected: 3.68 mm, 4.01 mm, 4.28 mm, and 4.44 mm. A total of n = 80 grains were measured using the TMS-PRO texture analyzer to perform uniaxial compression tests, yielding compression force-displacement curves for wheat grains during loading (Fig 13a-13d). The triaxial dimensions, mass, and compression force-displacement curve of each wheat grain were recorded in association with each other. Fig 14 shows the observed changes in the shape of the wheat particles during the compression process.

The specific fracture energy of wheat is calculated using Eq (16) based on the average force-displacement curve. The specific fracture energy is equal to the area under the force-displacement curve between the initial contact point and the crushing point of the particle divided by the corresponding particle mass:


$$E=\frac{\int_{\text{0}}^{\Delta c}Fd\Delta }{m_{\text{0}}}$$ (16)

where *F* is the compression force when the compression displacement is Δ, N; Δc is the displacement when wheat reaches the breaking point, mm; and Δ is the displacement of the press head during wheat compression, mm.

To process the data and calculate the effective experimental wheat particle-specific fracture energy *E* for each group, the wheat particle-specific fracture energy was statistically ranked, and the breakage probability was calculated using Eq (17):


$$P=\frac{i-\text{0}.\text{5}}{N}$$ (17)

where *i* is the serial number of wheat particles in ascending order of particle-specific fracture energy in the experimental sample; and *N* is the number of experimental samples.

**Fig 15:**
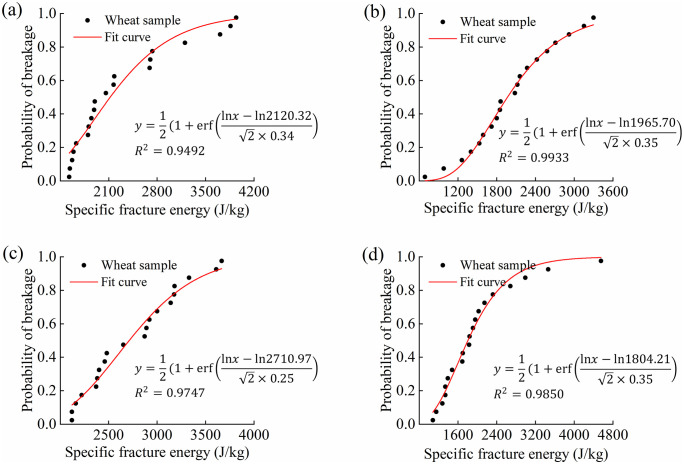
Probability of breakage of wheat grains as a function of specific fracture energy for different equivalent diameters: (a) 3.68 mm; (b) 4.01 mm; (c) 4.28 mm; (d) 4.44 mm. Note: The equation in the figure is the fitted log-normal distribution function (Eq 1), and R² indicates the goodness-of-fit.

The above data were calibrated using Eqs (1) and (2), and a fitting curve of the probability of breakage versus the specific fracture energy was plotted (Fig 15).

The median fracture energy E₅₀ and log-normal standard deviation σ for wheat grains at each equivalent diameter grade, obtained through curve fitting, are as follows: 3.68 mm grade: *E*₅₀ = 2710.97 J/kg, σ = 0.25; 4.01 mm grade: *E*₅₀ = 2120.32 J/kg, σ = 0.34; 4.28 mm grade: *E*₅₀ = 1965.70 J/kg, σ = 0.35; 4.44 mm grade: *E*₅₀ = 1804.21 J/kg, σ = 0.35.

To obtain representative E₅₀ values for the wheat sample population, weighted averages were calculated based on the distribution frequency of each grade within the natural population. Based on the size distribution statistics of 80 randomly selected wheat grains, the weights for the four grain grades (3.68 mm, 4.01 mm, 4.28 mm, and 4.44 mm) were 0.15, 0.28, 0.32, and 0.25, respectively. Applying these weights to the E₅₀ values of each grain grade yielded a weighted average *E*₅₀ of 2069.78 J/kg.

As shown in Fig 15 and Table 6, the smaller the particle size, the higher the fracture energy required for particle crushing. Nonlinear fitting of E₅₀ was performed via an iterative method combined with Eq (3), based on experimental data from different particle size ranges. The fitted model parameters were: *E*_∞_=73.40 J/kg, *d*₀ = 5.34 mm, φ = 12.63, with a correlation coefficient R² = 0.99192.

**Table 6:** Equivalent diameter, fracture energy, and sample size for different wheat particle size classes.

*L* (mm)	*W* (mm)	*T* (mm)	*D*_e_ (mm)	Sample size (*n*)	*E*_50_ (J/Kg)
5.6 (±0.2)	3.18 (±0.05)	2.79 (±0.05)	3.68	12	2710.97
6.0 (±0.2)	3.06 (±0.05)	3.49 (±0.05)	4.01	22	2120.32
6.4 (±0.2)	3.21 (±0.05)	3.78 (±0.05)	4.28	26	1965.70
6.8 (±0.2)	3.32 (±0.05)	3.86 (±0.05)	4.44	20	1804.21

#### 3.2.2 Wheat damage accumulation coefficient.

**Fig 16:**
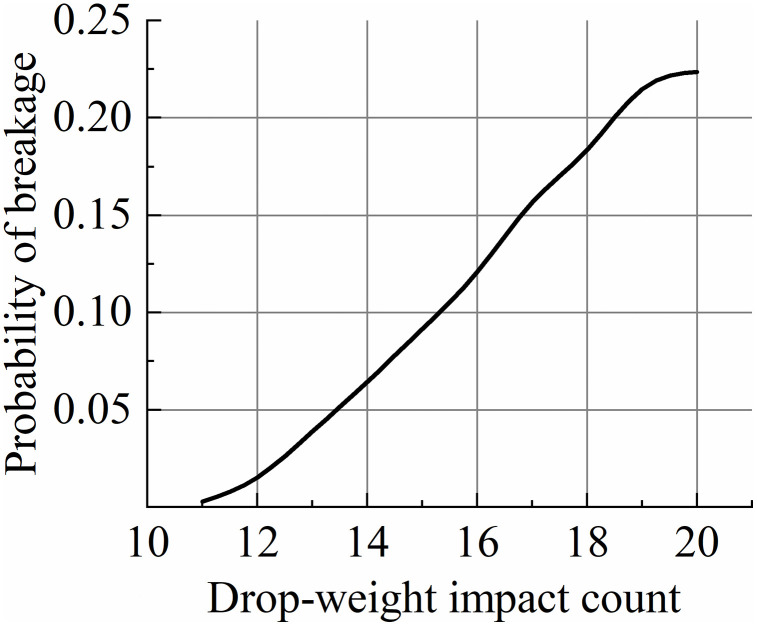
Relationship curve between wheat grain probability of breakage and drop-weight impact count.

The results of the multiple drop-weight impact tests are shown in Fig 16. This indicates that the probability of breakage of the wheat increased with an increase in the number of impacts. By combining the experimental results with Eqs (5) and (6) using parameters from the breakage probability model, the cumulative damage coefficient was estimated to be of 5 using the least squares method. γ is the only parameter in this model and must be determined based on data generated from repeatedly impacting particles at the applied energy level. *D* is implicitly defined in (6); therefore, the equation is iteratively solved starting from *D* = 0 [15].

### 3.3 Simulation compression test verification

The distribution of wheat fragments is related to the specific impact and fracture energies of the particles [32]. In this study, the relevant parameters from the wheat grain Ab-T10 fracture model calibrated by Zhang Chao et al. [8] were adopted, with *t*_10_ = 1.55% and Max *t*_10_ = 4.97%. The statistical results were fitted using Eq (9), yielding *A* = 50% and *b* = 0.1808, where parameter *A* is a general parameter [20]. The compression simulation test parameters for the Tavares wheat model are listed in Table 7. The simulation setting parameters are shown in Table 8.

**Table 7:** Simulation compression test parameters.

Performance and contact parameters			Tavares model parameters	
Wheat	Density (kg/m^3^)	3050	Damage constant γ	5
	Poisson ratio	0.42	Residual crushing energy *E*_∞_ (J/Kg)	73.40
	Shear modulus (Pa)	6.484 × 10^7^	Characteristic size of the microstructure *d*_0_ (mm)	5.34
Roller	Density (kg/m^3^)	7500	*E*_50_ as a power-law function of force intensity φ	12.63
	Poisson ratio	0.25	Standard deviation of the fracture energy σ	0.32
	Shear modulus (Pa)	4.5 × 10^10^	*A* (%)	50
Wheat-wheat	Elastic restitution coefficient	0.405	*b*	0.1808
	Static friction coefficient	0.322	Minimum particle size for breakage *D*_min_, (mm)	0.30
	Rolling friction coefficient	0.039	Minimum collision energy *E*_min_ (J/Kg)	0.0001
Wheat- Roller	Elastic restitution coefficient	0.399	Fraction of shear energy *C*_t_ (%)	0
	Static friction coefficient	0.426	Truncation ratio (%)	100
	Rolling friction coefficient	0.179	Max *t*_10_ (%)	4.97

**Table 8:** Simulation setting parameters.

Project	Value	Unit
Rayleigh percentage	0.01	%
Cell size	2	R_min_
Approx humber of cells	47432	
Compression speed	0.00025	m/s
Total time	10.2	s

**Fig 17:**
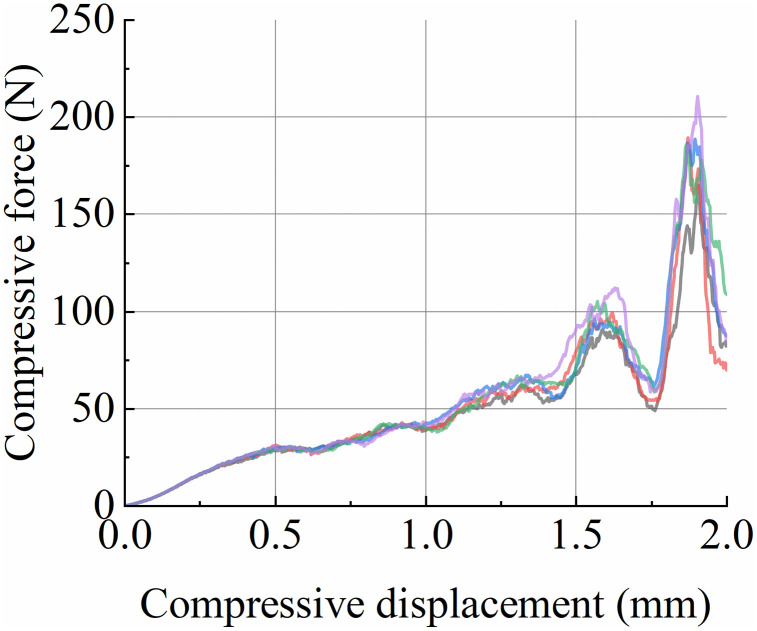
Simulated force-displacement curve.

Five simulated compression tests were conducted based on the calibrated parameters of the Tavares model for wheat. During the simulations, the particles were placed on a load-bearing plate, and a cylindrical indenter was used to apply compressive loading at a constant speed of 15 mm/min. After particle breakage, the compression force-time data were extracted, and force-displacement curves were plotted based on conversion using the indenter speed. The five replicated simulations produced force-displacement curves that nearly overlapped, as shown in Fig 17, demonstrating high consistency among the repeated simulations.To quantitatively evaluate the repeatability of the simulation results, descriptive statistical analysis was performed on the key fracture characteristic values extracted from the five independent simulation curves. For the peak fracture force, the mean value of the five replicates was 99.79 N, with a standard deviation (SD) of 0.41 N and a coefficient of variation (CV) of 0.41%. For the fracture energy at the fracture point, the mean value of the five replicates was 157.67 mJ, with an SD of 0.30 mJ and a CV of 0.19%. The extremely low coefficients of variation (all < 0.5%) quantitatively verify the excellent stability and repeatability of the calibrated Tavares model, and the high consistency of the simulation results further supports the reliability of the model calibration.

The calibration accuracy of the Tavares breakage model was preliminarily evaluated by comparing the ensemble-averaged simulated and experimental values of fracture force (*F*) and fracture energy (*E*′), as summarized in Table 9. The experimental values of *F* and *E*′ were 95.52 N and 155.70 mJ, while the simulated values were 99.79 N and 157.67 mJ, with relative errors of 4.47% and 1.27%, respectively. This mean-level comparison only confirms that the model predictions are statistically unbiased, with no systematic overestimation or underestimation of wheat fracture characteristics.

**Table 9:** Comparison of wheat grain breakage results.

Indicator	Parameters	
	Fracture force *F*/N	Fracture energy *E*′/ (mJ)
Experimental value	95.52	155.70
Simulated value	99.79	157.67
Relative error	4.47%	1.27%

However, we acknowledge the inherent limitation of this method: it cannot fully characterize the model’s forecast accuracy for the full compression force-displacement curve. Rigorous forecast accuracy testing requires statistical metrics including root mean square error (RMSE) and coefficient of determination (R²), which quantify point-by-point deviations between simulated and experimental curves. Owing to the discrete sampling of force-displacement data from the texture analyzer, we were unable to perform paired point-to-point calculation of these metrics in this study. Follow-up work will collect continuous high-frequency curve data to supplement this statistical validation and further optimize the model.

It is noteworthy that the model validation process is independent of the parameter calibration procedure: model calibration employs solely the fracture energy at the fracture point of uniaxial compression tests, whereas validation is conducted on the full-range force-displacement curve encompassing the entire compression fracture process. The calibrated model predicts the complete mechanical response of wheat grains under compression, rather than merely reproducing the parameter values used for fitting.

## 4 Discussion

### 4.1 Contact parameter interactions and model validation

The statistical results of the regression model (P < 0.0001) demonstrate that the model is statistically significant at the 0.1% level, and the non-significant lack-of-fit term (P = 0.1323) confirms that the quadratic model has a good fit to the experimental data. The 1.05% relative error between the simulated and experimental angle of repose reflects a high consistency between the two, confirming that the calibrated contact parameters can accurately characterize the inter-particle interactions of wheat. In addition, the R² value of 0.99192 for the nonlinear fitting of *E*₅₀ indicates the high reliability of the fitted model parameters.

The variation observed in the measured contact parameters (Table 1) reflects the inherent heterogeneity of wheat kernels as a biological material, with irregular shape and local property variations contributing to the within-group standard deviations. Despite this variability, the consistency of the mean values across repeated measurements confirms the reliability of the custom-built measurement apparatus.

The response surface analysis (Fig 12) reveals distinct roles of the three contact parameters in determining the macroscopic angle of repose. Static friction and rolling friction both exhibit strong positive effects, as increased resistance to sliding and rolling leads to steeper pile formation, and their significant synergistic interaction further amplifies this effect. This finding is highly consistent with the conclusions of Liu et al. [31] and Zhang et al. [29], who conducted DEM-based pile test studies on wheat and reported that frictional characteristics (rather than collision-related restitution) are the dominant factors governing the static packing behavior of wheat granular materials. Liu et al. [31] further pointed out that the rolling friction coefficient has a more pronounced effect on the angle of repose of wheat than the static friction coefficient, which is also verified in our study by the much higher F-value of rolling friction (F = 1345.62) than static friction (F = 266.61) in the regression model (Table 5). In contrast, the coefficient of elastic restitution shows negligible influence on the angle of repose, which is in line with the physical interpretation of quasi-static pile formation and is consistent with the experimental results of Wu et al. [27] on peanut seed particle DEM simulations, where the restitution coefficient was also found to have no statistical effect on the bulk stacking characteristics of agricultural granular materials.

The fracture energy of wheat grains was found to decrease with increasing particle equivalent diameter (Table 6), which conforms to the classic particle breakage energy scaling law in granular mechanics. This regularity is also observed in the Tavares model calibration studies of other agricultural particles: Shen et al. [15] reported a similar negative correlation between rice grain particle size and median specific fracture energy (E₅₀) in their rice breakage simulation based on the Tavares model, and Li et al. [7] also found that the fracture energy of polyhedral cotton seeds decreased with increasing particle size when calibrating the Tavares model parameters. Our study further quantified this scaling relationship for wheat via nonlinear fitting (R² = 0.99192), and the obtained power-law exponent (φ = 12.63) reflects the unique mechanical response of wheat’s ellipsoidal anisotropic structure—compared with the nearly spherical rice grains (φ = 8.21, Shen et al. [15]), wheat requires a larger exponent due to the layered bran-endosperm structure, which makes the particle size effect on fracture energy more significant.

Comparison of simulated and experimental force-displacement curves (Fig 17) demonstrates that the calibrated Tavares model successfully reproduces the key mechanical responses of wheat under compression, including the initial elastic regime, yielding, crack propagation, and final fracture. The relative errors of fracture force (4.47%) and fracture energy (1.27%) between simulation and experiment are significantly lower than those of the Ab-T10 wheat fracture model calibrated by Zhang et al. [8] (relative errors of 8.9% and 7.3% for fracture force and energy, respectively), which verifies the superiority of the Tavares model in characterizing wheat breakage behavior. This advantage is mainly attributed to the Tavares model’s consideration of damage accumulation and fracture energy log-normal distribution, which makes it more suitable for simulating the complex stress environment of roller milling than the Ab-T10 model that is only applicable to specific impact scenarios. In addition, the calibration accuracy of our wheat Tavares model is comparable to that of the cotton seed Tavares model established by Li et al. [7] (relative error of fracture force <5%), which further proves the universality of the Tavares model in the breakage simulation of agricultural granular materials with different morphological and structural characteristics.

However, a systematic deviation is observed in the slope of the elastic region, where the simulated response is slightly less stiff than the experimental curve. This discrepancy likely arises from the model's simplification of wheat as a homogeneous material, neglecting the anisotropic structure of the endosperm and bran layers that contributes to initial stiffness. Additionally, the exclusion of fine progeny particles from the computational domain after fracture may affect the post-peak response. Nevertheless, the close agreement in fracture force and fracture energy confirms that the model captures the dominant energy dissipation mechanisms during compression. The calibrated parameters thus provide a sound foundation for simulating wheat milling processes, where accurate prediction of fracture initiation and energy consumption is critical.

### 4.2 Discussion of experimental limitations

This study, centered on the core goal of establishing parameter correlations between experiments and simulations, adopts a simplified assumption of homogeneous and isotropic material properties for wheat grains. This simplification exhibits practical validity in the context of agricultural granular material engineering simulations.Wang et al. [33] demonstrated that variations in the material properties of wheat grains along different orientations had a negligible impact on simulation outcomes. By employing Box-Behnken design and response surface analysis, they validated that a simplified parameter calibration method based on the homogeneity assumption can accurately predict the mechanical behaviors of wheat grains. Liu et al. [34] demonstrated via comparative analysis of anisotropic and isotropic models in simulations of grain ventilation systems that anisotropic effects were relatively weak in wheat grain materials. The isotropic assumption was shown to meet the accuracy requirements for most agricultural engineering simulations.

However, the inherent properties of natural wheat grains may still affect simulation accuracy. The heterogeneity of wheat grains originates from biological individuality; even after uniform conditioning, subtle differences persist in the microstructure and component distribution of individual grains, rendering complete uniformity in physical and mechanical properties challenging. Anisotropy, by contrast, is associated with wheat’s ellipsoidal geometry, the stratified structure of the endosperm and aleurone layer, and the oriented arrangement of internal starch granules and cell walls, which imparts pronounced directional dependence to its mechanical behavior.

These characteristics primarily affect simulation outcomes in three key aspects: (1) Heterogeneity induces variations in deformation resistance and fracture sensitivity among particles, leading to deviations between simulated local stress distributions and actual conditions. Although this may alter the fracture sequence of individual particles, its influence on macro-statistical fracture patterns remains limited; (2) Anisotropy results in wheat particles displaying distinct force responses under different grinding orientations. This leads to simulated force-displacement curves that lack the natural granularity observed in experiments, hindering the accurate reproduction of actual fracture paths and fragment morphologies; (3) The calibration of contact parameters and the angle of repose is based on the average behavior of randomly oriented particles, overlooking orientation-dependent variations. This may introduce slight deviations, yet the model remains reliable for engineering simulation purposes.

Future refinements will involve direction-dependent parameter assignment, integrate microstructure reconstruction techniques, and implement multi-orientation calibration experiments to improve simulation precision. The simplifying assumptions of the current model do not undermine its core objectives and continue to provide effective theoretical support for optimizing wheat milling processes and equipment design.

Furthermore, the characterization of wheat grain samples in this study primarily relied on measurements of geometric dimensions (length, width, thickness) and equivalent diameter, with no differential analysis performed on the intrinsic quality attributes of individual grains (including hardness, vitreous rate, and moisture distribution). In reality, the mechanical behavior of wheat grains is closely correlated with their intrinsic quality. Future research will utilize a Single Kernel Characterization System (SKCS) for the systematic characterization of samples. The SKCS 4100 model (Perten Instruments, Sweden) is capable of simultaneously measuring the mass, diameter, hardness, and moisture content of individual wheat grains. This facilitates the provision of more precise input parameters for discrete element models, particularly through the establishment of a direct correlation between grain hardness distribution and fracture energy distribution in the Tavares model. This further enhances the model's capacity to characterize grain heterogeneity and anisotropy, thereby improving the accuracy and generalizability of simulation results.

### 4.3 Optimising industrial milling with calibrated DEM simulations

The precise calibration of the Tavares model parameters for wheat established here extends beyond validation, providing a reliable theoretical foundation for optimising industrial grinding through DEM simulation. Specifically, the calibrated parameters enable: (1) virtual testing of mill designs (e.g., roll gap settings and feed rates), reducing development time and cost before prototyping; (2) prediction of the particle size distribution of ground material under different operational conditions, directly linking process to product quality; and (3) estimation of milling forces and energy expenditure via DEM, facilitating energy efficiency analysis and the identification of ways to lower specific energy consumption.

Given the typical condition-dependent mechanical and contact behaviors of biological granular materials such as wheat, the calibrated DEM parameters in this study have specific application boundaries, while the established multi-dimensional parameter calibration system features universal transferability. For different wheat varieties, the calibrated contact and Tavares model parameters are partially transferable to other hard wheat varieties with similar ellipsoidal morphology and bran-endosperm structure, where only fracture energy-related parameters need fine recalibration due to varietal differences in endosperm vitreousness and bran thickness, but are not directly transferable to soft or durum wheat that differ greatly in intrinsic mechanical properties. For wheat moisture contents, the parameters are limitedly transferable within the industrial optimal moistening range of 15.5%–17.5% (only contact friction coefficients need fine-tuning by ±0.02), yet invalid for moisture contents outside this range due to drastic changes in wheat brittleness, viscoelasticity and surface adhesion, requiring full re-calibration. For milling conditions, the parameters are fully transferable to conventional white cast iron roller milling (300–500 r/min, 0.1–0.5 mm roll gap); for modified conditions such as altered roller materials or speeds, only wheat-roller contact parameters need re-measurement via the custom mechatronic device [26], while wheat intrinsic fracture parameters (fracture energy, damage coefficient) remain transferable, as equipment conditions only affect contact interactions rather than grain internal mechanical properties. Notably, extreme milling conditions (ultra-narrow roll gap < 0.1 mm, high speed >800 r/min) will change wheat stress states from compression-impact to shear-crushing, requiring supplementary calibration of shear fracture parameters based on the existing Tavares model framework. In summary, while the specific calibrated parameter values are condition-specific to Zhoumai 22 (16.32% moisture) and conventional roller milling, the calibration method established in this study is universally applicable to all wheat milling simulation scenarios, which is a key methodological contribution of this work.

## 5 Conclusion

This study addresses the lack of precise models for discrete element simulations of wheat compression and crushing processes, focusing on the parameter calibration and validation of the Tavares crushing model. Through a self-designed mechatronic measurement apparatus, combined with physical experiments and numerical simulation methods, the contact parameters between wheat grains and the grinding roller surface material (white cast iron), as well as those between wheat grains themselves, were calibrated. The calibrated contact parameters were validated using angle of repose tests, where the simulation results showed high consistency with the physical experimental outcomes, confirming the reliability of this set of contact parameters.

Through uniaxial compression tests and drop-weight impact tests, we systematically calibrated the key parameters of the Tavares model, clarifying the distribution characteristics of wheat’s fracture energy and specific fracture energy, and acquiring the model’s core mechanical parameters. Based on these findings, a Tavares model for wheat compression fracture was established. A comparative analysis was conducted between the simulated wheat compression force-displacement curve and actual experimental results, and the consistent response behaviors of the two confirmed the accuracy and reliability of the calibrated model parameters.

The calibrated Tavares model has clear predictive capability for wheat breakage behavior under different processing conditions, which provides a reliable numerical simulation tool for the research of wheat milling mechanism, optimization of milling process parameters, and roller mill structure design, and has important guiding value for improving the quality and efficiency of industrial wheat processing.

## Supporting information

S1 File Static friction coefficient measurements. (XLSX)

S2 File Rolling friction coefficient measurements. (XLSX)

S3 File Coefficient of restitution measurements. (XLSX)

S4 File Data underlying Fig_13a. (XLSX)

S5 File Data underlying Fig_15a. (XLSX)

S6 File Data underlying for Fig_16. (XLSX)

S7 File Simulation results. (XLSX)

S1 Abstract Graphical abstract. (TIF)
