# Supplementary figures and images for: Discrete element method parameter calibration and validation of wheat based on the Tavares model
Source: PLoS One. 2026 Apr 13;21(4):e0346715. doi: 10.1371/journal.pone.0346715 (PMC13075715; doi:10.1371/journal.pone.0346715)

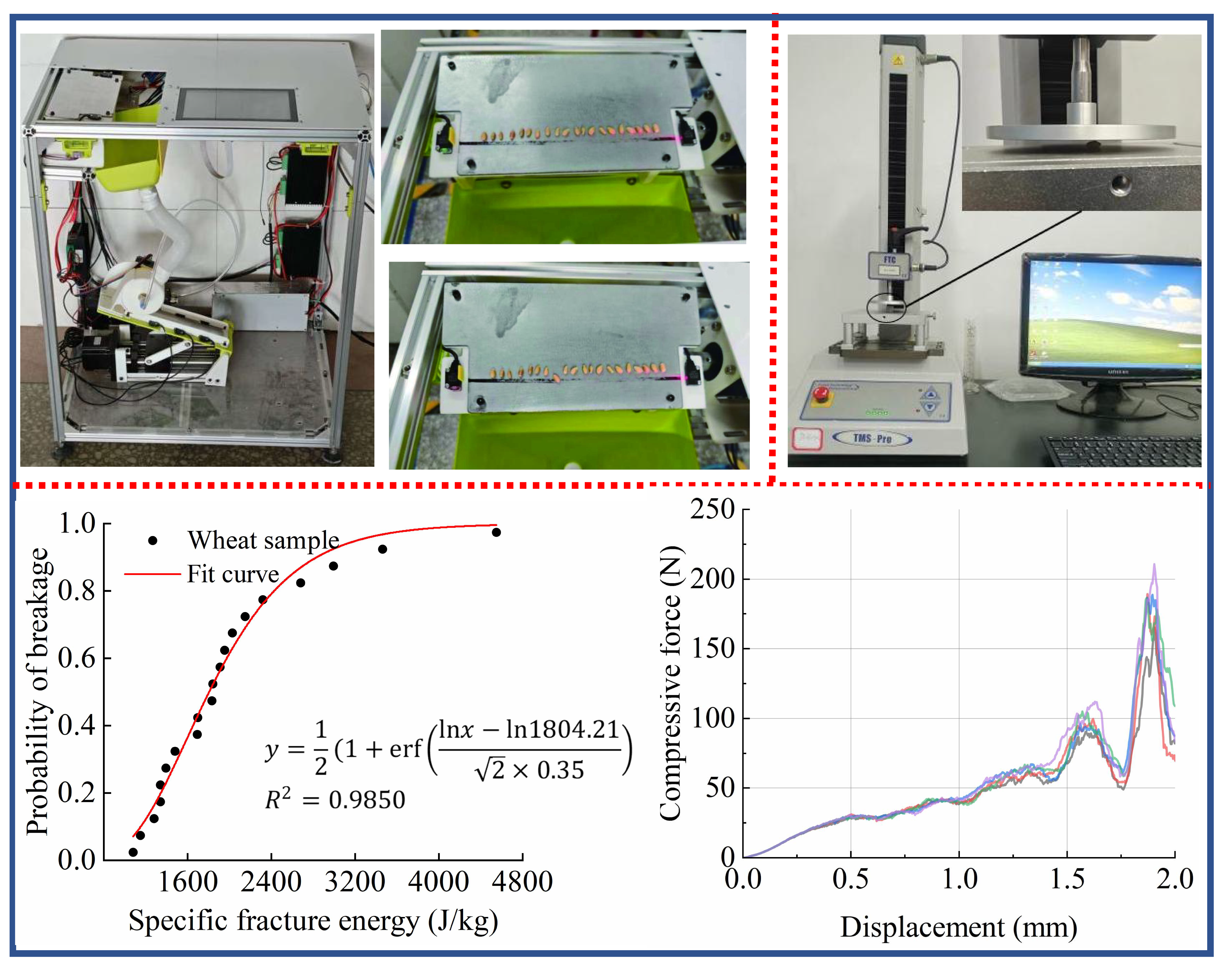

Supplement: S1 Abstract — (TIF) [file pone.0346715.s008.tif]
